# The efficacy, safety, and rationale of orelabrutinib combined with obinutuzumab (O2) as a first-line systemic treatment for marginal zone lymphoma

**DOI:** 10.1038/s41408-026-01575-y

**Published:** 2026-07-11

**Authors:** Jiadai Xu, Jing Li, Yuhong Ren, Luya Cheng, Zhixiang Cheng, Jingli Zhuang, Yang Ke, Shiyang Gu, Yian Zhang, Feifei Chen, Rupan Gao, Panpan Li, Yaqin Xiong, Peng Liu

**Affiliations:** 1 Department of Hematology, Zhongshan Hospital, Fudan University, Shanghai, China; 2 Cancer Center, Zhongshan Hospital, Fudan University, Shanghai, China

**Keywords:** B-cell lymphoma, Targeted therapies

**To the Editor:**

Marginal zone lymphoma (MZL) is an indolent B-cell malignancy characterized by protracted clinical courses, frequent relapses, and significant biological heterogeneity. The current frontline standard of care relies heavily on rituximab-based immunochemotherapy. While these regimens effectively induce remissions, they are intrinsically linked to cumulative toxicities—including neuropathy, prolonged myelosuppression, and the latent risk of secondary malignancies—that compromise long-term quality of life [1, 2]. Consequently, defining highly efficacy, chemotherapy-free alternatives remain a critical unmet need in previously systematic untreated MZL patients.

Bruton’s tyrosine kinase (BTK) inhibitors have fundamentally altered the therapeutic landscape for relapsed or refractory MZL [3]. However, attempts to pair first-generation BTK inhibitors such as ibrutinib with anti-CD20 antibody–based regimens have produced inconsistent results [4, 5]. We therefore reasoned that a more selective BTK inhibitor, devoid of the off-target effects that may compromise innate immune effector function, might be combined more productively with a potent anti-CD20 antibody—a hypothesis we revisit mechanistically below.

Orelabrutinib is a novel, highly selective BTK inhibitor with minimal off-target kinase activity [6]. Obinutuzumab, a glycoengineered type II anti-CD20 antibody, induces markedly superior direct B-cell killing and ADCC compared to rituximab [7]. We hypothesized that combining orelabrutinib with obinutuzumab (the O2 regimen) would construct a rational, chemotherapy-free frontline platform. By selectively disabling B-cell receptor (BCR) signaling without paralyzing the innate immune system, this combination should permit the enhanced ADCC properties of obinutuzumab to operate unhindered.

To test this, we conducted a prospective observational cohort study of the O2 regimen in 26 patients with newly diagnosed MZL requiring systemic therapy (20 MALT, 2 nodal, 1 splenic, and 3 undetermined subtypes). This study was exploratory in nature and all consecutive eligible patients during the study period were enrolled. Patients were eligible if they were ≥18 years of age, had histologically confirmed CD20-positive MZL, and had progressed/relapsed after or were ineligible for local therapy. Additional key inclusion criteria were an Eastern Cooperative Oncology Group (ECOG) performance status of 0–2, life expectancy of ≥3 months, and adequate hematologic and organ function. Key exclusion criteria included refusal to use reliable methods of contraception during reproductive age or during lactation, presence of severe mental illness, and/or determination of ineligibility by the investigator. This trial was conducted in compliance with the Declaration of Helsinki and the International Conference on Harmonisation Guidelines for Good Clinical Practice and was approved by the institutional review board of Zhongshan Hospital Affiliated to Fudan University (No. B2023-184). All patients provided written informed consent before enrollment. No identifiable images of human participants are included in this study. This study was registered at ClinicalTrials.gov under the identifier number NCT06203652.

Patients received six 28-day induction cycles of oral orelabrutinib (150 mg/day) and intravenous obinutuzumab (1000 mg on Days 1, 8, and 15 of Cycle 1; Day 1 of Cycles 2–6) (Fig. 1A); responders then transitioned to orelabrutinib maintenance for up to one year. Tumor response was evaluated using Lugano 2014 or GELA criteria [8, 9].

**Fig. 1:**
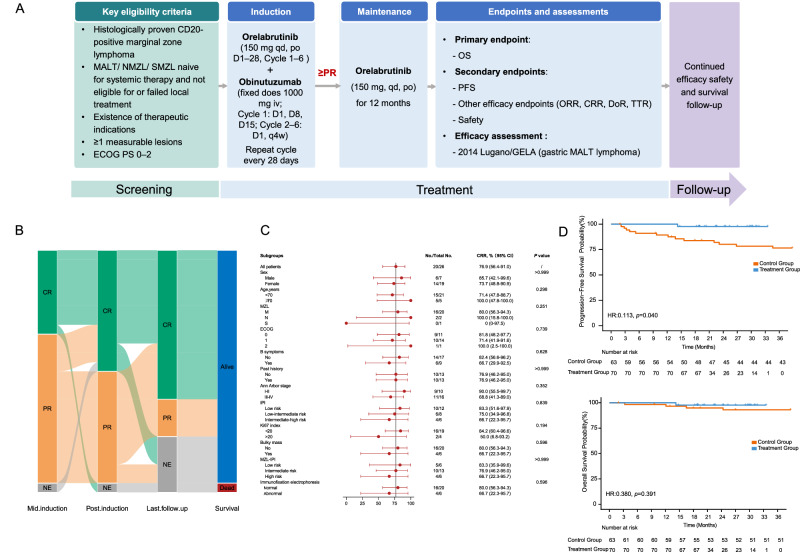
**A** Overview of the study design. **B** Alluvial plot depicting 5 patients who achieved PR after induction treatment subsequently achieved CR during maintenance treatment. **C** Subgroup analysis of CRR in the O2 cohort. **D** Comparison of PFS and OS between the O2 group and the control group. Abbreviations: CI confidence interval, CR complete response, CRR complete response rate, DoR duration of response, ECOG Eastern Cooperative Oncology Group, IPI International Prognostic Index, MZL marginal zone lymphoma, NE not evaluated, OS overall survival, O2 orelabrutinib plus obinutuzumab, PFS progression-free survival, PR partial response.

While randomized controlled trials remain the gold standard for comparative efficacy, such designs are difficult to execute in early-phase development for heterogeneous, indolent lymphomas. To estimate efficacy while mitigating the selection bias inherent to single-arm data, we applied inverse probability of treatment weighting (IPTW), weighting the O2 cohort against an institutional historical control of 52 MZL patients treated with standard immunochemotherapy (R-CHOP, R-COP, or bendamustine-rituximab). Drawing the control arm from the same institution minimizes the geographic, temporal, and supportive-care confounding inherent to comparisons with external registries.

MZL comprises distinct clinical entities; consistent with its epidemiology, MALT lymphoma was the predominant subtype (76.9%). The cohort represented a challenging demographic: median age 61.0 years, 61.5% with advanced Ann Arbor Stage III–IV disease, and 76.9% with intermediate or high-risk MZL-IPI scores. All 26 patients completed induction. At a median follow-up of 24.4 months, the best overall response rate (ORR) was 100% and the complete response rate (CRR) ultimately reached 76.9%. This deep remission accrued progressively: the post-induction CRR was 53.8%, and five patients with an initial partial response (PR) converted to a CR during single agent orelabrutinib maintenance (Fig. 1B). Subgroup analyses showed that adverse prognostic markers—advanced stage, high tumor burden, elevated Ki67, and intermediate/high-risk IPI [10]—did not attenuate response depth (Fig. 1C). Median time to initial response was 3.0 months and to CR 5.9 months. The 24-month progression-free survival (PFS) and overall survival (OS) were both 96.2% (Fig. 1D). After IPTW adjustment, the adjusted ORR was significantly higher than immunochemotherapy (100% versus 86.9%, *p* = 0.014); the CRR was numerically higher (82.5% versus 68.2%) but not significantly so, and PFS was significantly improved (HR 0.113, *p* = 0.040) (Fig. 1D).

While long-term durability data are required to confirm survival benefits in indolent lymphomas, the depth of early response—particularly a complete response—is a robust surrogate for prolonged progression-free survival. Achieving a 76.9% CRR in the frontline setting without cytotoxic agents establishes a favorable foundation for durable disease control.

Equally important in the frontline management of an incurable lymphoma is the toxicity profile. The safety of the O2 regimen was highly manageable and compared favorably to the known toxicities of standard chemotherapy. Sixteen patients (61.5%) experienced at least one adverse event (AE). Grade 3 or 4 AEs were remarkably limited, occurring in four patients (15.4%), and consisted entirely of uncomplicated neutropenia. Grade 1 or 2 pneumonitis occurred in six patients (23.1%). Notably, these events occurred predominantly during maintenance (5/6), while patients were taking oral orelabrutinib at home, and most represented community-acquired pneumonia. They clustered in winter and spring. Together with the humoral immunodeficiency that follows obinutuzumab- and orelabrutinib-induced B-cell depletion, this pattern indicates increased susceptibility to seasonal respiratory infection, rather than direct pulmonary toxicity, as the principal contributor. All events were low grade and resolved with anti-infective therapy—most commonly fluoroquinolones such as moxifloxacin—without permanent discontinuation, the O2 regimen being continued or resumed in every case. Continued vigilance for respiratory infection, with attention to vaccination and immunoglobulin levels, remains advisable. We did not observe a single instance of the severe cardiovascular toxicities (such as atrial fibrillation) or major hemorrhagic events that routinely complicate the use of earlier BTK inhibitors. No grade 5 AEs occurred. The tolerability of this regimen is perhaps best evidenced by the fact that only one patient discontinued maintenance therapy due to a drug-related AE (grade 1 gingival bleeding).

To link these clinical observations with tumor biology, we performed Visium high-definition spatial transcriptomics (ST) on MALToma biopsies from three patients, with cell-type annotations validated by multiplex immunofluorescence (mIF) (Supplemental Fig. S1A, B) [11]. Highly proliferative (MKI67-high) malignant B-cells showed upregulation of NF-κB, FcγR-mediated phagocytosis, and BCR signaling pathways (Supplemental Fig. S1C). The only patient with just a partial response during induction (a thyroid MALToma) showed a distinct deficiency of natural killer (NK) T cells. Spatially resolved neighborhood analyses (50-μm and 100-μm zones) demonstrated varying B-cell and NKT-cell colocalization (Supplemental Fig. S1D–G), suggesting that the density and proximity of functional NKT cells are rate-limiting for anti-CD20-mediated tumor clearance. The *AddModuleScore* algorithm confirmed that NKT cells harbored the highest ADCC scores within the microenvironment (Supplemental Fig. S1H). This dual-activation profile supports our strategy of dismantling BCR/NF-κB signaling via orelabrutinib while exploiting FcγR phagocytosis via obinutuzumab.

We functionally validated this synergy stepwise. Using engineered Jurkat reporter cells expressing high-affinity (V158) or low-affinity (F158) FcγRIIIa co-cultured with B-cell lymphoma lines (TMD-8 and REC-1), obinutuzumab elicited superior baseline ADCC compared with rituximab [7]. As anticipated, ibrutinib markedly inhibited, and zanubrutinib moderately reduced, obinutuzumab-mediated ADCC in both high-affinity (Fig. 2A) and low-affinity (Fig. 2C) assays, whereas orelabrutinib preserved it. The same pattern—preservation by orelabrutinib versus inhibition by earlier-generation inhibitors—was seen with rituximab in high-affinity (Fig. 2B) and low-affinity (Fig. 2D) systems.

**Fig. 2:**
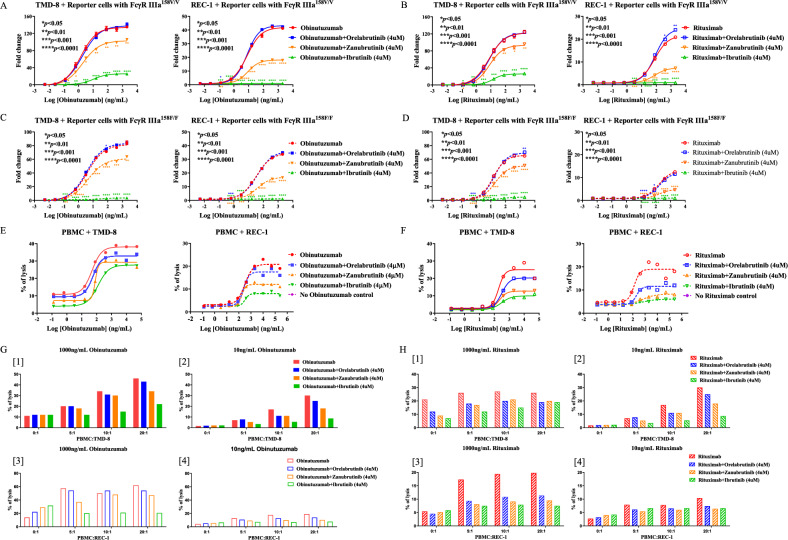
Effect of BTK inhibitors on ADCC activity. **A** The impact of the 3 BTK inhibitors for obinutuzumab in high-affinity Jurkat FcγRIIIa158V/V cells co-cultured with TMD-8 or REC-1 cells. **B** The impact of the 3 BTK inhibitors for rituximab in high-affinity Jurkat FcγRIIIa158V/V cells co-cultured with TMD-8 or REC-1 cells. **C** The impact of the 3 BTK inhibitors for obinutuzumab in low-affinity Jurkat FcγRIIIa158F/F cells co-cultured with TMD-8 or REC-1 cells. **D** The impact of the 3 BTK inhibitors for rituximab in low-affinity Jurkat FcγRIIIa158F/F cells co-cultured with TMD-8 or REC-1 cells. **E** For obinutuzumab, in the co-culture with REC-1 cells, the effect of orelabrutinib, zanubrutinib, and ibrutinib on the ADCC bioactivity induced by PMBC-derived NK cells. **F** For rituximab, in the co-culture with REC-1 cells, the effect of orelabrutinib, zanubrutinib, and ibrutinib on the ADCC bioactivity induced by PBMC-derived NK cells. **G** The impact of obinutuzumab at different concentrations and E:T ratios, in combination with the 3 BTK inhibitors, on the induction of lysis in TMD-8 ([1] and [2]) and REC-1 ([3] and [4]) tumor cells. **H** The impact of rituximab at different concentrations and E:T ratios, in combination with the 3 BTK inhibitors, on the induction of lysis in TMD-8 ([1] and [2]) and REC-1 ([3] and [4]) tumor cells. Abbreviations: ADCC antibody-dependent cellular cytotoxicity, BTK Bruton’s tyrosine kinase, E:T effector-to-target, NK natural killer, PBMC peripheral blood mononuclear cell.

In a more physiological setting, we performed co-culture assays with primary human PBMC-derived NK cells. Dose-response analyses of the half-maximal effective concentration (EC50) and maximum effect (Emax) confirmed that orelabrutinib spared primary NK-cell activity, whereas ibrutinib and zanubrutinib blunted it (Fig. 2E, F), and orelabrutinib maintained obinutuzumab-induced target-cell lysis across escalating effector-to-target (E:T) ratios (Fig. 2G, H). Thus, orelabrutinib’s strict kinase selectivity protects FcγR-mediated activation and primary NK-cell function, allowing obinutuzumab’s enhanced ADCC to operate without drug-induced immune interference [12].

These findings help reconcile an apparent paradox in the literature on BTK inhibitor/anti-CD20 antibody combinations. On one hand, ibrutinib can antagonize rituximab-dependent, NK cell–mediated cytotoxicity through irreversible interleukin-2-inducible T-cell kinase (ITK) inhibition [13], and in the randomized Alliance A041202 trial adding rituximab to ibrutinib did not improve PFS in chronic lymphocytic leukemia [14]; conversely, combinations such as ibrutinib plus obinutuzumab (iLLUMINATE [15]) and orelabrutinib plus rituximab [12] have shown clear benefit. Our data suggests this discrepancy relates to the off-target kinase profile of the agent used, with greater kinase selectivity—particularly minimal ITK inhibition—being more compatible with preserved NK-cell, antibody-dependent effector function. Thus, the pharmacological profile of the individual inhibitor, rather than BTK inhibition itself, may determine its compatibility with an effector-dependent anti-CD20 antibody. Although derived from limited in vitro assays that warrant further confirmation, these observations provide a mechanistic rationale for pairing the highly selective orelabrutinib with the glycoengineered, ADCC-enhanced antibody obinutuzumab.

While the single-arm design, the modest sample size, and the reliance on a weighted historical comparison rather than randomization or a formal matching-adjusted indirect comparison pose inherent limitations, the integration of prospective outcomes, IPTW benchmarking, and spatially resolved mechanistic validation makes a coherent case. The O2 regimen provided deep, durable responses with a tolerable safety profile in treatment-naïve MZL. By uncoupling BCR inhibition from the suppression of innate ADCC, it circumvents the failures of prior BTKi/anti-CD20 combinations and merits definitive evaluation in randomized clinical trials.

## Supplementary information

Supplemental material

Figure S1

Figure S2

## Data Availability

The raw Visium high-definition spatial transcriptomics data generated during the current study and the processed gene expression data are available in the Gene Expression Omnibus (GEO) repository under accession number [GSE337521]. The clinical data that supports the findings of this study are available from the corresponding author upon reasonable request.
