# Supplementary material for: The efficacy, safety, and rationale of orelabrutinib combined with obinutuzumab (O2) as a first-line systemic treatment for marginal zone lymphoma
Source: Blood Cancer J. 2026 Jul 11;16(1):114. doi: 10.1038/s41408-026-01575-y (PMC13356022; doi:10.1038/s41408-026-01575-y)
Supplement: Supplementary file 1 — Supplemental material [file 41408_2026_1575_MOESM1_ESM.docx]

**Supplemental Materials**

**Supplemental Methods.**

**Analysis of cell resolution spatial transcriptomics (ST) using Visium high-definition (HD) technology**

#### With the introduction of Visium HD technology (10x Genomics, Pleasanton, CA) enabling the study of cell resolution ST, we analyzed Visium HD spatial gene data from 3 patients with mucosa-associated lymphoid tissue lymphoma (MALToma) who had different treatment responses to the orelabrutinib plus obinutuzumab (O2) regimen in this cohort, to explore the key molecular pathways, critical cell subpopulations, communication patterns, and spatial distributions that are potentially correlated with O2 therapeutic sensitivity.

#### *Visium HD ST tissue sectioning*

Formalin-fixed paraffin-embedded (FFPE) sections from 3 patients with MALToma (1 gastric MALToma, 1 thyroid MALToma, and 1 labial gland MALToma) were collected from the O2 cohort and subjected to Visium HD ST (**Figure 1A**). First, 5-µm sections of the 3 samples were taken from the FFPE tissue blocks using a microtome (RWD Life Science Co., Ltd, Shenzhen, China). Five-µm FFPE sections were placed on capture areas of the immunocytochemistry slides, incubated for 2 hours at 42°C, then allowed to dry at room temperature.

#### *Visium HD spatial gene expression*

The isolation of total RNA from FFPE tissue blocks was achieved using the RNeasy FFPE kit (73504, Qiagen, Hilden, Germany). The RNA quality assessment was performed by calculating DV200 (percentage of RNA fragments >200 nucleotides). Tissue sections passing the quality control (DV200 >30%) were subjected to ST assay. The Visium HD workflow requires new HD slides that need thawing, washing, and equilibration in appropriate buffers. Visium HD slides also feature high-resolution fiducials for sub-pixel image alignment, and a dispensing pad and spacer for CytAssist compatibility. We first placed FFPE tissue sections on plain glass slides for deparaffinization, hematoxylin and eosin staining, and imaging following the Visium HD FFPE Tissue Preparation Handbook (CG000684). Subsequently, the sections were stained with hematoxylin and eosin and imaged at 20 × magnification in brightfield using a PANNORAMIC MIDI II Digital Scanner (3DHISTECH, Budapest, Hungary). Probe hybridization, probe ligation, slide preparation, probe release, extension, library construction, and sequencing followed the Visium HD Spatial Gene Expression Reagent Kits User Guide (CG000685). Sequencing was performed on an Illumina (San Diego, CA) NovaSeq 6000 with paired-end reads (43 cycles Read 1, 10 cycles i7, 10 cycles i5, 50 cycles Read 2). Space Ranger v3.0 was used to map FASTQ files to the human reference, detect the tissue section, align the sequencing data to the microscope image and the CytAssist image, and output gene-barcode matrices for further analysis.

#### *Identification of cell types in Visium HD spatial and downstream analysis*

Seurat objects (version 5.2.0) were initialized for each sample using the 8 × 8 µm filtered feature barcode matrices generated by Space Ranger. For thyroid MALToma and gastric MALToma samples, HD bins with fewer than 50 unique molecular identifier (UMI) counts or exceeding 25% mitochondrial expressions were filtered out. The raw counts were independently normalized using log-normalization. Clustering of the Visium HD data was conducted within Seurat. The initial clustering round identified clusters at a resolution of 0.3. We then annotated the predominant cell types within each cluster using marker genes and refined our annotations by re-clustering the B-cell clusters. For the labial gland MALToma samples, HD bins with fewer than 25 UMI counts or over 25% mitochondrial expression were excluded. Following this, cell-type deconvolution was performed on each Visium HD 8-µm bin from the same region using SPOTlight, with single-cell RNA sequencing (scRNA-seq) data serving as the reference.

For the analysis of cell communication, CellChat (version 2.1.2) was used with several key parameter settings. To mitigate the impact of outliers and ensure more robust results, we trimmed 10% of the data from both ends and calculated the mean of the remaining 80%. Spatial distances between cells were considered to incorporate spatial context in cell communication analysis, and the interaction range for cell communication was set to 20 µm. Additionally, cell groups consisting of fewer than 5 cells were filtered out to prevent unreliable inferences.

#### *Analysis of the B cells and plasma B cells at various distances from the natural killer T (NKT) cell periphery*

To evaluate the potential functions of these cells, we used the AddModuleScore function from Seurat (version 5.2.0) and performed pairwise comparisons using the Wilcoxon test. The results were visualized using ggplot2 for plotting and heatmap for heatmap generation.

For differential analysis, we used the FindMarkers function in Seurat (version 5.2.0). The screening criteria for significant genes were *p* <0.01, |log2FC| ≥0.26 (corresponding to a fold change of at least 1.2 times), and expression in at least 10% of the cells. Kyoto Encyclopedia of Genes and Genomes analyses were performed using the OmicStudio cloud platform provided by LC-Bio Technologies (Hangzhou, China).^1^ The analysis of NKT cells at various distances from the periphery of B cells or plasma B cells was conducted in a similar way.

#### *ScRNA-seq and downstream analysis*

Although Visium HD enables cell resolution ST analysis, the labial gland MALToma sample showed relatively low mean unique molecular identifier counts per 8-µm bin compared with the other 2 samples. Therefore, scRNA-seq was added to compensate for the limited information obtained from Visium HD.

scRNA-seq results were demultiplexed and converted to FASTQ format using Illumina bcl2fastq software (version 5.0.1). Sample demultiplexing, barcode processing, and gene counting were performed with Cell Ranger (version 7.2.0), and scRNA-seq data were aligned to the Ensembl genome GRCm39 reference genome. The pipelines were run with the assistance of LC-Bio Technologies.

We performed downstream analysis using the Seurat package (version 4.4.1). Cells with fewer than 400 detected genes and cells with mitochondrial transcript percentages <25% were excluded from the analysis. Additionally, we used the DoubletFinder software (version 2.0.4) to remove potential doublets. After filtering out low-quality cells and doublets, the data were normalized using the NormalizeData function, high-variable genes were identified with the FindVariableFeatures function, and the data were scaled using the ScaleData function. Subsequently, dimensionality reduction was performed using RunPCA for principal component analysis. Clustering was conducted using the FindNeighbors and FindClusters functions. Finally, the clustering results were visualized using DimPlot.

The FindAllMarkers function was used to identify characteristic genes for each cluster. Subsequently, we performed enrichment analysis on the characteristic genes of each cluster using the cluster Profiler R package (version 4.12.6) with default settings. The human annotation information was sourced from the org.Hs.eg.db database. Additionally, cell-type identification and annotation were based on the following markers: acinar cells (PRSS1, CTRB1, CTRB2, REG1A), plasma cells (MZB1, JCHAIN, XBP1), B cells (MS4A1, CD79B), myeloid cells (LYZ, CD68), fibroblast cells (DCN, COL1A1, COL1A2), NKT cells (CD3D, CD3E), epithelial cells (KRT4, KRT13), and endothelial cells (PECAM1, VWF, CDH5).

We used CellChat (2.1.2), a database that can accurately analyze cell–cell signal connections and build communication networks, to explore potential cell–cell interactions in the lymphoma microenvironment. By using the default parameters and the ligand–receptor pair database, if cell type A expresses the ligand that is paired with the receptor expressed by cell type B, then the event is regarded as a valid interaction. According to the molecular characteristics of the ligand–receptor pair, the interaction events are divided into secretory signals, cell–cell contact, and extracellular matrix receptors. We demonstrated the potential communication relationships between interested cell types. Communication events between different cell types were displayed in the form of connecting lines.

We used the AddModuleScore from the Seurat package to evaluate the potential function of each cell subtype. This function returned a module score for each cell, reflecting the relative enrichment of a pre-set gene set (module) compared with a background gene set, thereby quantifying the activity level of biological processes or signaling pathways in individual cells.

#### *Multiplex immunofluorescence based on the tyramide signal amplification (TSA) system*

Paraffin sections were baked at 62°C for 1 h, deparaffinized in xylene (3 × 5 min), and hydrated through graded ethanol (70%–100%). Antigen retrieval was performed using ethylenediaminetetraacetic acid buffer (microwave heating, 30 min at 95°C). After phosphate-buffered saline (PBS) washes, autofluorescence was quenched with a photo-bleaching reagent. For each staining cycle, slices were incubated with primary antibodies (e.g., CD56, CD20, CD138; diluted as specified) at 37°C for 20 min, followed by horseradish peroxidase-conjugated secondary antibodies (37°C, 20 min). TSA was performed using CF 488, Cy3, or AF 647 tyramide (room temperature, 6 min). Antibody stripping (37°C, 20 min) and PBS washes preceded subsequent rounds. 4′,6-diamidino-2-phenylindole counterstaining (5 min) and mounting were conducted after each cycle. Three sequential staining rounds (R1–R3) were executed, with fluorescence quenching between cycles. The final slides were scanned using a digital slide scanner (DS-Fi2; Nikon, Tokyo, Japan), and images were merged for analysis.

#### *Cell cultures and reagents*

Jurkat-NFAT-Luc2-CD16-F158/V158 cell lines were purchased from Kyinno Biotechnology (Beijing, China). Healthy donor peripheral blood mononuclear cells (PBMCs) were sourced from Shanghai Yayu Biological Medicine (Shanghai, China). TMD-8 and REC-1 cell lines were purchased from Nanjing Cobioer Biosciences (Nanjing, China). Cryopreserved cells were maintained in RPMI 1640 Medium GlutaMAX supplement (Thermo Fisher, Waltham, MA), supplemented with 10% heat-inactivated fetal bovine serum (FBS; Genetimes, Hong Kong, China). Cells were incubated at 37°C in a 5% CO_2_ humidified environment. Rituximab and obinutuzumab were provided by Roche (Basel, Switzerland), orelabrutinib was gifted by InnoCare Pharma (Beijing, China), and ibrutinib and zanubrutinib were purchased from Selleckchem (Houston, TX).

#### *Antibody-dependent cell-mediated cytotoxicity (ADCC) reporter bioassay*

TMD-8 and REC-1 cell lines were incubated at a 1:1 ratio with FcgRIIIa/NFAT-Luc2 (luciferase reporter gene luc2)-expressing Jurkat cell lines (Jurkat-NFAT-Luc2-CD16-F158/V158 cell lines) for 6 hours at 37°C and in 5% CO_2_ in the presence/absence of rituximab or obinutuzumab using 5-fold serial dilutions. Luciferase activity was quantified using the Bright-Glo (Promega, Madison, WI) reagent, and luminescence was measured using the microplate reader BioTeK Synergy H1 (Agilent, Santa Clara, CA). Data were expressed as luminescence (relative light unit; RLU) fold of induction = RLU induced (rituximab or obinutuzumab alone, or in combination with BTK inhibitor)/RLU no antibody control.

#### *PBMC-derived NK cell-mediated ADCC assay*

TMD-8 and REC-1 cell lines were labeled with 1.6 µM of carboxyfluorescein succinimidyl ester (CFSE, Thermo Fisher) for 10 minutes in 1 mL of PBS. A 10-fold volume of culture medium with 10% FBS was added for 20 minutes to quench the reaction, and cells were washed 3 times. CFSE-labeled cells were then counted and incubated together with 4 µM of compounds at a concentration of 2 × 10^6^/mL in RPMI 1640 medium supplemented with 10% FBS for 1 hour. Treated target cells were then co-cultured together with PBMCs for 5 hours at 4:1 effector-to-target (E:T) ratios, in the presence of 5-fold serial dilutions of rituximab or obinutuzumab. Rituximab (5 μg/mL)-treated cells were used as a negative control. Samples were collected and acquired with a flow cytometer (FACS, Agilent), and data were analyzed with the built-in FACS analysis software. Cell lysis was calculated using Viability Dye eFuor 506 (Thermo Fisher) as a cell death marker. ADCC efficiency was expressed as adjusted lysis, calculated as 100-survival, namely 100 × (survival with effectors/survival without effectors).

#### *Cell apoptosis*

Healthy volunteer-derived PBMC cells (sourced from Shanghai Yayu Biological Medicine, Shanghai, China) were pre-incubated with kinase inhibitors for 1 hour. TMD-8 and REC-1 cells were then transduced and labeled with CFSE. Obinutuzumab, rituximab, or PBS was added to wells containing the PBMC cells and lymphoma cells, which were subsequently co-incubated for 60 hours at 37°C. Following incubation, cells were stained with propidium iodide and Annexin-5. Apoptosis was quantified using a flow cytometer (Agilent).

***Endpoint definitions***

Progression-free survival was calculated from the time of treatment initiation to the first disease progression or death from any cause, whichever occurred first. Overall response rate was defined as the proportion of patients with complete response (CR) or partial response. Complete response rate refers to the proportion of patients with CR. Duration of response was the time from the first evidence of response until disease progression or death from any cause, whichever occurred first. Time to response was calculated from the date of initiation of therapy to the time of response.

**Reference**

1. Lyu F, Han F, Ge C, et al. OmicStudio: A composable bioinformatics cloud platform with real-time feedback that can generate high-quality graphs for publication. *Imeta*. 2023;2(1):e85.

**Supplemental Results**

**Supplemental Results S1.** Obinutuzumab demonstrated superior anti-CD20-antibody-induced antibody-dependent cellular cytotoxicity (ADCC) activity compared with rituximab in the reporter bioassay. Both obinutuzumab and rituximab induced strong ADCC activity against diffuse large B-cell lymphoma (DLBCL) TMD-8 cells when co-cultured with high-affinity Jurkat FcγRIIIa^158V/V^ cells, with half maximal effective concentration (EC_50_) values of 1.44 ng/mL and 7.83 ng/mL, and maximum effect (E_max_) values of 134% and 122%, respectively. In contrast, when co-cultured with mantle cell lymphoma (MCL) REC-1 cells, the ADCC activity induced by these antibodies was slightly lower, with EC_50_ values of 7.91 ng/mL and 75.59 ng/mL, and E_max_ values of 42% and 23%, respectively. Similarly, when using low-affinity Jurkat FcγRIIIa158F/F cells, obinutuzumab and rituximab induced strong ADCC activity against DLBCL TMD-8 cells, with EC_50_ values of 3.99 ng/mL and 17.33 ng/mL, and E_max_ values of 82% and 70%, respectively. Against MCL REC-1 cells, ADCC activity was slightly reduced, with EC_50_ values of 38.61 ng/mL and 170.7 ng/mL, and E_max_ values of 36% and 12%, respectively.

**Supplemental Results S2.** Obinutuzumab demonstrated superior anti-CD20-antibody-induced ADCC activity over rituximab in ADCC bioactivity induced by PBMC-derived natural killer cells. In the co-culture of PBMCs with TMD-8 cells, obinutuzumab achieved a half maximal effective concentration (EC_50_) of 48.9 ng/mL and a maximum effect (E_max_) value of 38%, outperforming rituximab, which exhibited an EC_50_ of 190.3 ng/mL and an E_max_ of 25%. In the co-culture with REC-1, obinutuzumab maintained higher ADCC activity with an EC_50_ of 310.4 ng/mL and an E_max_ of 21% compared with rituximab (EC_50_ of 128.8 ng/mL; E_max_ of 19%). For rituximab in the co-culture system of PBMC with TMD-8, the EC_50_ was 313.4 ng/mL with an E_max_ of 25%. In the co-culture of PBMC with the REC-1 system, rituximab had an EC_50_ of 128.8 ng/mL with an E_max_ of 19%. Zanubrutinib, and ibrutinib showed no significant effect over the vehicle control (18%) at 18%, 17%, and 13%, respectively (**Figure S2C**). Rituximab in REC-1 cells increased apoptosis to 53% with orelabrutinib, more effectively than zanubrutinib (48%) and ibrutinib (32%), with similar minimal effects over the vehicle control as seen in other figures (**Figure S2D**). Collectively, obinutuzumab was more potent than rituximab in inducing apoptosis in both MCL and DLBCL cell lines, with orelabrutinib consistently and significantly enhancing the apoptotic effect of both antibodies, thus highlighting its potential as an adjunct to improve cancer therapeutic efficacy by boosting immune-mediated tumor cell apoptosis.

**Table S1.** Baseline characteristics in the O2 group and historical control group before and after IPTW

| **Characteristics** | **Before** **IPTW** | | | | **After IPTW** | | | |
| --- | --- | --- | --- | --- | --- | --- | --- | --- |
|  | **O2 group (N=26)** | **Control group (N=52)** | **SMD** | ***p*** | **O2 group (N=69.6)** | **Control group (N=63.3)** | **SMD** | ***p*** |
| Sex |  |  | 0.987 | **<0.001** |  |  | 0.121 | 0.725 |
| Male | 7 (26.9) | 37 (71.2) |  |  | 41.8 (60.1) | 41.8 (66.0) |  |  |
| Female | 19 (73.1) | 15 (28.8) |  |  | 27.7 (39.9) | 21.5 (34.0) |  |  |
| Median age at diagnosis (years) |  |  | 0.159 | 0.393 |  |  | 0.383 | 0.356 |
| Mean±SD | 59.5±13.3 | 57.4±12.2 |  |  | 63.28± 14.09 | 58.27± 12.03 |  |  |
| Histological type |  |  | 0.532 | 0.064 |  |  | 0.360 | 0.481 |
| M | 20 (76.9) | 46 (88.5) |  |  | 62.3 (89.5) | 56.1 (88.6) |  |  |
| N | 2 (7.7) | 5 (9.6) |  |  | 2.7 (3.8) | 5.6 (8.8) |  |  |
| S | 1 (3.8) | 1 (1.9) |  |  | 1.6 (2.3) | 1.6 (2.6) |  |  |
| Unknown | 3 (11.5) | 0 |  |  | 3.0 (4.3) | 0 |  |  |
| ECOG PS |  |  | 1.667 | **<0.001** |  |  | 0.632 | 0.369 |
| 0 | 11 (42.3) | 0 |  |  | 11.0 (15.8) | 0 |  |  |
| 1 | 14 (53.8) | 26 (50.0) |  |  | 37.8 (54.4) | 36.3 (57.3) |  |  |
| 2 | 1 (3.8) | 26 (50.0) |  |  | 20.8 (29.8) | 27.0 (42.7) |  |  |
| B symptoms |  |  | 0.082 | 0.732 |  |  | 0.608 | 0.095 |
| No | 17 (65.4) | 36 (69.2) |  |  | 30.5 (43.9) | 45.9 (72.6) |  |  |
| Yes | 9 (34.6) | 16 (30.8) |  |  | 39.1 (56.1) | 17.4 (27.4) |  |  |
| Past medical history |  |  | 0.038 | 0.873 |  |  | 0.270 | 0.438 |
| No | 13 (50.0) | 25 (48.1) |  |  | 24.9 (35.8) | 31.0 (49.0) |  |  |
| Yes | 13 (50.0) | 27 (51.9) |  |  | 44.7 (64.2) | 32.3 (51.0) |  |  |
| Ann Arbor stage |  |  | 0.248 | 0.298 |  |  | 0.096 | 0.787 |
| I–II | 10 (38.5) | 14 (26.9) |  |  | 25.7 (37.0) | 20.5 (32.4) |  |  |
| III–IV | 16 (61.5) |  |  |  | 43.9 (63.0) | 42.8 (67.6) |  |  |
| IPI score |  |  | 0.650 | 0.130 |  |  | 0.683 | 0.188 |
| 0–1 (low risk) | 12 (46.2) | 21 (40.4) |  |  | 28.4 (40.7) | 27.7 (43.8) |  |  |
| 2 (low–intermediate risk) | 8 (30.8) | 13 (25.0) |  |  | 29.7 (42.6) | 14.7 (23.3) |  |  |
| 3 (intermediate–high risk) | 6 (23.1) | 9 (17.3) |  |  | 11.6 (16.6) | 11.3 (17.9) |  |  |
| 4–5 (high risk) | 0 | 9 (17.3) |  |  | 0 | 9.5 (15.0) |  |  |
| Ki67 index |  |  | 0.298 | 0.478 |  |  | 0.646 | 0.072 |
| ≤20 | 19 (73.1) | 34 (65.4) |  |  | 56.3 (80.8) | 38.8 (61.4) |  |  |
| >20 | 4 (15.4) | 14 (26.9) |  |  | 4.6 (6.6) | 19.3 (30.5) |  |  |
| Unknown | 3 (11.5) | 4 (7.7) |  |  | 8.7 (12.5) | 5.2 (8.2) |  |  |
| Bulky disease |  |  | 0.132 | 0.588 |  |  | 0.256 | 0.412 |
| No | 20 (76.9) | 37 (71.2) |  |  | 58.7 (84.3) | 46.9 (74.0) |  |  |
| Yes | 6 (23.1) | 15 (28.8) |  |  | 10.9 (15.7) | 16.4 (26.0) |  |  |
| MALT-IPI score |  |  | 0.298 | 0.459 |  |  | 0.531 | 0.339 |
| 0 | 10 (38.5) | 13 (25.0) |  |  | 25.7 (37.0) | 18.3 (28.9) |  |  |
| 1 | 11 (42.3) | 28 (53.8) |  |  | 18.9 (27.2) | 32.5 (51.4) |  |  |
| 2 | 5 (19.2) | 11 (21.2) |  |  | 25.0 (35.9) | 12.4 (19.7) |  |  |
| MZL-IPI score |  |  | 0.439 | 0.258 |  |  | 0.882 | 0.055 |
| 0 (low risk) | 6 (23.1) | 15 (28.8) |  |  | 21.3 (30.7) | 18.6 (29.4) |  |  |
| 1–2 (intermediate risk) | 13 (50.0) | 31 (59.6) |  |  | 19.4 (27.8) | 38.6 (60.9) |  |  |
| 3–5 (high risk) | 7 (26.9) | 6 (11.5) |  |  | 28.9 (41.5) | 6.1 (9.7) |  |  |
| Previous local therapy |  |  | 0.340 | 0.210 |  |  | 0.168 | 0.561 |
| No | 19 (73.1) | 45 (86.5) |  |  | 56.0 (80.5) | 54.9 (86.7) |  |  |
| Yes | 7 (26.9) | 7 (13.5) |  |  | 13.6 (19.5) | 8.4 (13.3) |  |  |
| Anti-Helicobacter pylori therapy | 2 (28.6) | 0 |  |  | 7.1 (52.2) | 0 |  |  |
| Surgery | 5 (71.4) | 7 (100.0) |  |  | 6.5 (47.8) | 8.4 (100.0) |  |  |

Data are expressed as n (%) or median (range).

Abbreviations: IPTW, inverse probability of treatment weighting; M, mucosa-associated lymphoid tissue; N, nodal marginal zone lymphoma; S, splenic marginal zone lymphoma; IPI, International Prognostic Index; ECOG PS, Eastern Cooperative Oncology Group Performance Status; MALT, mucosa-associated lymphoid tissue; MZL, marginal-zone lymphoma; O2, orelabrutinib + obinutuzumab; SMD, standardized mean difference.

**Table S2.** Best overall response between the O2 group and the historical control group

| **Parameter** | **Before IPTW** | | **After IPTW** | | |
| --- | --- | --- | --- | --- | --- |
|  | **O2 group (N=25)** | **Control group (N=51)** | **O2 group** | **Control group** | ***p*** |
| ORR | 26 (100.0) | 45 (88.2) | 69.6 (100.0) | 54.1 (86.9) | **0.014** |
| CR/CRu | 20 (76.9) | 34 (66.7) | 57.4 (82.5) | 42.5 (68.2) | 0.290 |
| PR | 6 (23.1) | 11 (21.6) | 12.1 (17.5) | 11.6 (18.7) | 0.915 |
| SD | 0 | 2 (3.9) | 0 | 3.7 (5.9) |  |
| PD | 0 | 4 (7.8) | 0 | 4.5 (7.2) |  |

Data are expressed as n (%).

Abbreviations: CR, complete response; CRu, unconfirmed complete response; IPTW, inverse probability of treatment weighting; ORR, overall response rate; O2, orelabrutinib + obinutuzumab; PD, progressive disease; PR, partial response; SD, stable disease.

**Figure Legends**

**Figure S1. Visium HD ST analysis of B cells and NKT cells from gastric MALToma, labial gland MALToma, and thyroid MALT lymphoma.**

(A) Different primary cell types in the 3 MZL samples, and the percentage of each primary cell type in the 3 samples. (B) The spatial cell-type annotation from Visium HD ST demonstrated strong agreement with mIF-based validation in tissue sections, supporting the accuracy of transcriptomic immune cell identification. (C) The differentially expressed genes in B cells and plasma B cells stratified by MKI67 expression level across the 3 samples. (D) B cells and plasma B cells at various distances from NKT cells (specifically: 50-µm B cells and 50-µm plasma B cells, 100-µm B cells and 100-µm plasma B cells, and B cells and plasma B cells including 50-µm B cells [proximal zone], 100-µm B cells [proximal zone], and B-cell populations located at distances >100 µm). (E) Percentage of each B-cell distance-related subtype from the 3 samples. (F) Heatmap visualization revealed distinct pathway activation patterns across the spatial subgroups. (G) Results from Visium HD ST; NKT cells were classified by various distances from B cells or plasma B cells, into 50-µm NKT cells, 100-µm NKT cells, and remote NKT cells located at distances >100 µm. (H) The AddModuleScore algorithm was used to calculate ADCC scores for each cell subpopulation.

Abbreviations: ADCC, antibody-dependent cellular cytotoxicity; HD, high-definition; KEGG, Kyoto Encyclopedia of Genes and Genomes; MALT, mucosa-associated lymphoid tissue; MALToma, mucosa-associated lymphoid tissue lymphoma; mIF, multiplex immunofluorescence; MZL, marginal zone lymphoma; NKT, natural killer T; ST, spatial transcriptomics.

**Figure S2. Obinutuzumab demonstrated superior anti-CD20-antibody-induced ADCC activity over rituximab in ADCC bioactivity induced by PBMC-derived natural killer cells.**
