# Supplementary figures and images for: The efficacy, safety, and rationale of orelabrutinib combined with obinutuzumab (O2) as a first-line systemic treatment for marginal zone lymphoma
Source: Blood Cancer J. 2026 Jul 11;16(1):114. doi: 10.1038/s41408-026-01575-y (PMC13356022; doi:10.1038/s41408-026-01575-y)

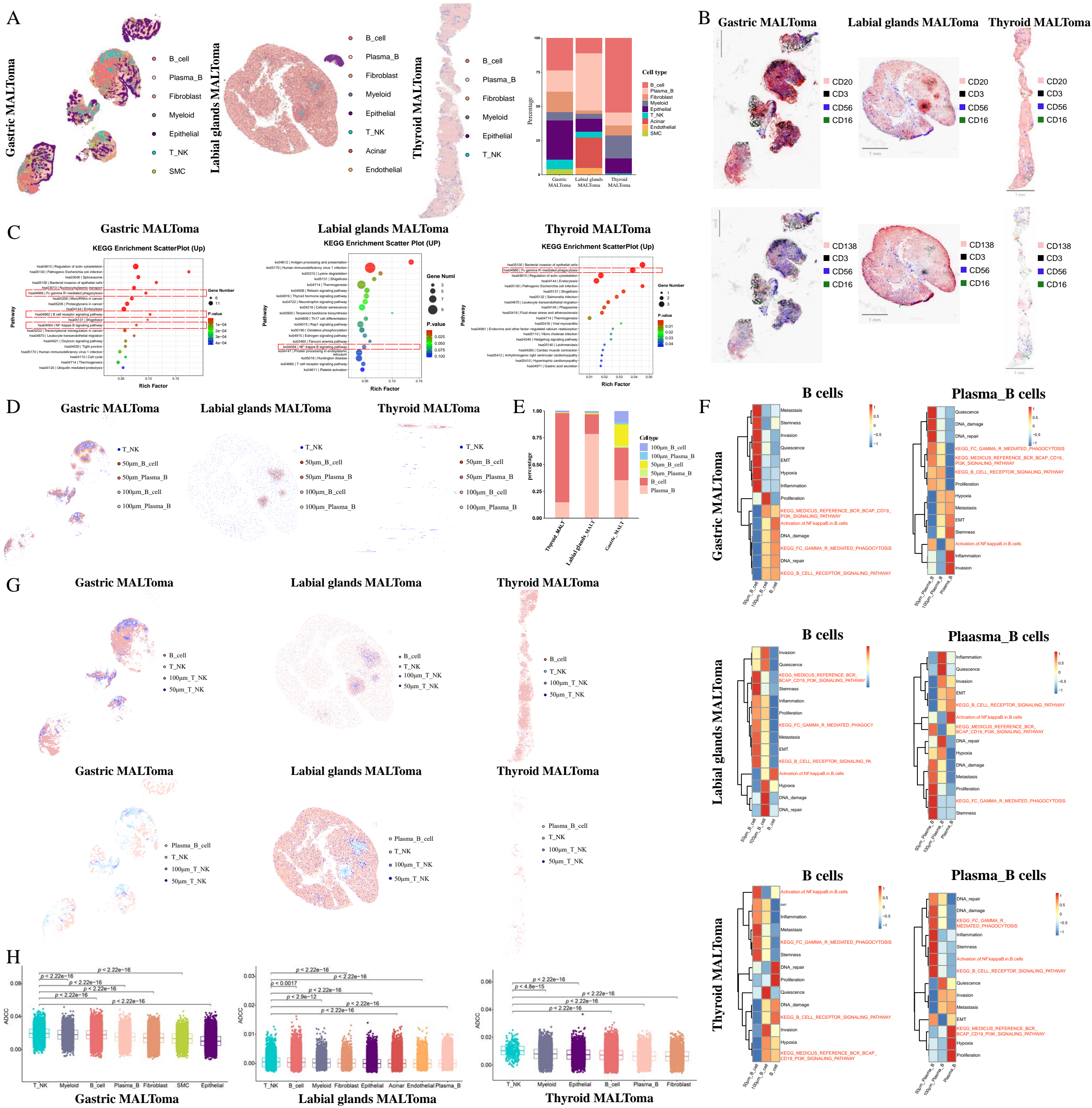

Supplement: Supplementary file 2 — Figure S1 [file 41408_2026_1575_MOESM2_ESM.pdf]

**A****TMD-8 + PBMC**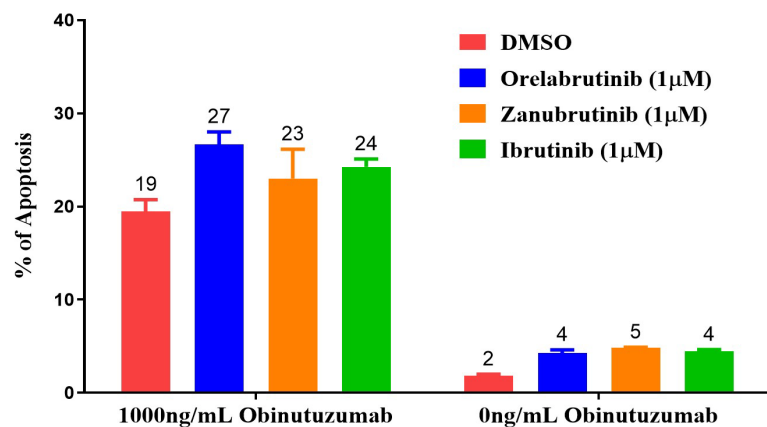**B****TMD-8 + PBMC**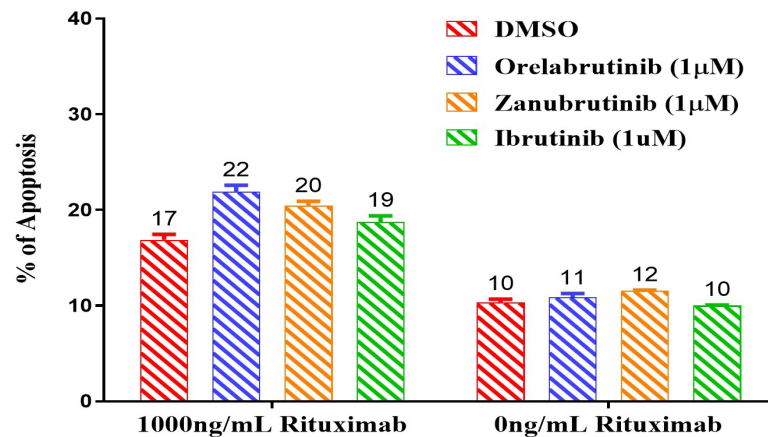**C****REC-1 + PBMC**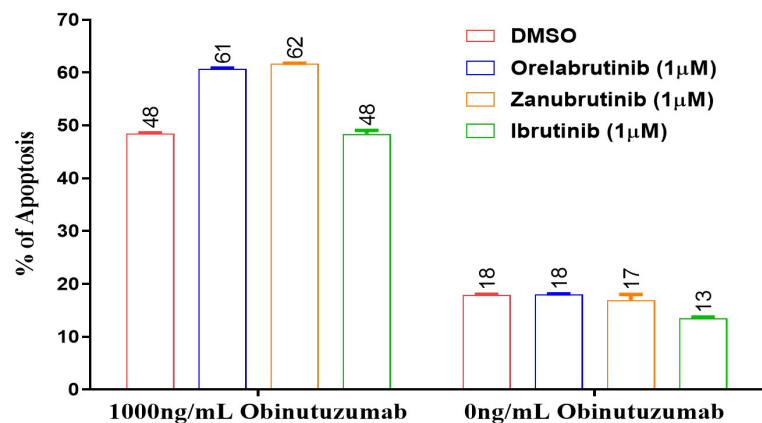**D****REC-1 + PBMC**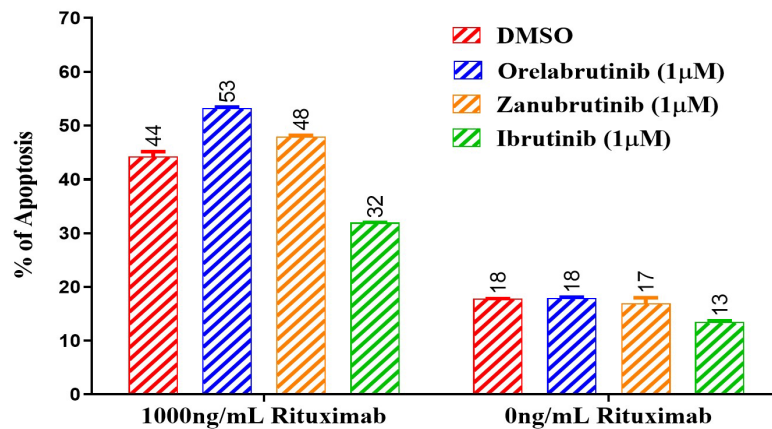

Supplement: Supplementary file 3 — Figure S2 [file 41408_2026_1575_MOESM3_ESM.pdf]
